# Mixed-Input Deep Learning Approach to Sleep/Wake State Classification by Using EEG Signals

**DOI:** 10.3390/diagnostics13142358

**Published:** 2023-07-13

**Authors:** Md. Nazmul Hasan, Insoo Koo

**Affiliations:** Department of Electrical, Electronic and Computer Engineering, University of Ulsan, Ulsan 44610, Republic of Korea; hasan01@mail.ulsan.ac.kr

**Keywords:** sleep–wake classification, mixed-input model, EEG, deep learning, sleep stages

## Abstract

Sleep stage classification plays a pivotal role in predicting and diagnosing numerous health issues from human sleep data. Manual sleep staging requires human expertise, which is occasionally prone to error and variation. In recent times, availability of polysomnography data has aided progress in automatic sleep-stage classification. In this paper, a hybrid deep learning model is proposed for classifying sleep and wake states based on a single-channel electroencephalogram (EEG) signal. The model combines an artificial neural network (ANN) and a convolutional neural network (CNN) trained using mixed-input features. The ANN makes use of statistical features calculated from EEG epochs, and the CNN operates on Hilbert spectrum images generated during each epoch. The proposed method is assessed using single-channel Pz-Oz EEG signals from the Sleep-EDF database Expanded. The classification performance on four randomly selected individuals shows that the proposed model can achieve accuracy of around 96% in classifying between sleep and wake states from EEG recordings.

## 1. Introduction

Sleep is essential for maintaining both mental and physical health. This biological process helps the body recover and rejuvenate at the end of the day. Bad quality sleep has numerous adverse physiological effects that lead to many short-term and long-term consequences: stress, somatic problems, obesity, type 2 diabetes, and even death [1]. However, research shows it is possible to obtain valuable insight into a person’s present and future health issues by analyzing sleep patterns. A study from the American Academy of Sleep Medicine (AASM) revealed it is possible to predict future health issues like dementia, cardiovascular health, psychological disorders, and mortality by analyzing EEG signals recorded during sleep [2]. Research from the University of California, Berkeley, conducted on 32 healthy adults confirmed that studying sleep patterns can predict development of Alzheimer’s disease [3]. Sleep-related research covers a wide spectrum. With the availability of different physiological signal databases and data processing tools and algorithms, more and more researchers with expertise in signal processing and artificial intelligence are extracting significant insights from data related to sleep. Among sleep research topics, sleep stage classification is important for understanding the structure of sleep and for diagnosing and treating sleep disorders, monitoring sleep quality, and improving overall sleep health. Classification of sleep stages is generally carried out using physiological signals from the electroencephalogram (EEG), electrocardiogram (ECG), electrooculogram (EOG), and electromyogram (EMG), and from pulse oximetry, respiratory signals, body temperature, and thoracic and abdominal movements, etc. Trained experts analyze these signals and designate a specific sleep stage for each 30 s of a recording or epoch. Generally, there are two standards for labeling sleep stages: the Rechtschaffen and Kales (R&K) rule [4] and a form of R&K modified by the AASM [5].

According to the R&K rules, EEG patterns are divided into seven different stages: wake, stage 1, stage 2, stage 3, stage 4, stage REM (rapid eye movement), and movement time. These labels are assigned to the EEG recordings by expert technicians observing other physiological signals such as eye movements, muscle activity, and electrooculography (EOG) simultaneously. Since this method heavily depends upon visual analysis and subjective interpretation of sleep recordings, it is prone to subjectivity and inter-expert reliability. Additionally, R&K rules were developed for young healthy adults. Thus, it is not directly applicable to elderly subjects and patients [6]. On the other hand, the American Academy of Sleep Medicine (AASM) proposes a more comprehensive and standardized modification of the R&K standard. The AASM divides sleep into three non-rapid eye movement (NREM) stages (N1, N2, N3) and one rapid eye movement (REM) stage. This method provides detailed scoring criteria for each sleep stage based on the presence or absence of specific waveforms, frequencies, and amplitudes in the EEG, as well as features such as eye movements and muscle tone. The AASM guidelines specify criteria for determining the onset and offset of sleep. Different types of movements, such as periodic limb movements or excessive body movements, are identified and scored separately. The AASM scoring manual includes guidelines for separately scoring respiratory events, such as apneas and hypopneas. The AASM guidelines also address technical considerations related to sleep recording and scoring, including the use of appropriate montages, filters, and sampling rates. It also provides recommendations for artifact identification and removal to ensure accurate and reliable sleep stage scoring.

Assigning labels to sleep epochs by visually scrutinizing polysomnography signals is a complex and challenging process that requires careful observations by an expert technician. Expert systems, that rely on rule-based algorithms to classify sleep stages following specific criteria and decision thresholds determined by experts’ guidelines, are used for sleep stage classification. As such an approach involves human involvement, it is a time-consuming task and, due to the chaotic nature and variability of the signals, there is the possibility of different observers interpreting the same epoch differently. Therefore, research to develop an automatic sleep stage classification system with considerable accuracy is important to ease the process and assist technicians involved in such tasks. Emergence of artificial intelligence (AI) aided the automatic sleep stage classification greatly. Combined with signal processing, deep learning and machine learning algorithms are now able to classify sleep stages with satisfactory accuracy without expert involvement [7,8].

## 2. Related Works

In recent times, automated sleep stage classification has been largely based on machine learning and deep learning frameworks as data collection, processing, and sharing become more feasible [9,10]. Sleep stage classification based on different polysomnography data has been explored in some research [11,12,13]. Polysomnography signals collected from wearable sensors have also been used for sleep stage detection owing to the ease of use and low cost. Wrist-worn accelerometer data were used for sleep–wake state detection in [14] with random forest as the classifier model. A decision-tree-based classifier was implemented in [15] using features computed from hand acceleration, ECG signals, and distal skin temperature collected by a wearable, wireless sensor. The model’s ability to detect sleep and wake states is higher than from using other wearable sensor data. It has been observed that among the signals used for diagnosing sleep, the EEG is considered by a majority of researchers for its high temporal accuracy, non-invasiveness, the availability of wearable sensors, and ease of use [16].

In [17], the authors provided an extensive comparison of common machine learning algorithms and neural network performance for automatic sleep stage classification using EEG signals from the Sleep-EDF expanded database. At the pre-processing stage, DC artifacts and low-frequency deviations are removed, which is followed by a denoising step where two independent components are denoised using a wavelet transform. Later, several features are extracted from the reconstructed denoised signal in four broad categories: time-based, frequency-based, entropy-based, and non-linear features. Based on all these features, support vector machine (SVM), random forest (RF), and multi-layer perceptron (MLP) classifiers provided accuracy of 94%. The authors also discussed the effect on classification accuracy from selecting a single-channel and a multi-channel EEG signal combination. A similar approach was followed in [18] using a single-lead ECG signal labeled according to three sleep stages: wake, non-rapid eye movement (NREM), and rapid eye movement (REM). In that work, a total of 39 features were computed utilizing Singular Value Decomposition (SVD), Variational Mode Decomposition (VMD), Hilbert–Huang Transform (HHT), and a morphological method. Selective features using a wrapper approach attained good accuracy from the RF classifier. Sleep staging from EOG signals based on various statistical features extracted from detailed and approximate coefficients and obtained with a discrete wavelet transform (DWT) was investigated in [19]. The statistical features were grouped as spectral entropy-based, moment-based measures, refined composite multiscale dispersion entropy, and auto-regressive model coefficients. The authors summarized the classification performance when the output sleep stage varied from two-class to six-class, and their proposed scheme showed good accuracy when the SVM was used as the classifier.

The convolutional neural network (CNN) for its automatic feature extraction ability was used in conjugation with image-based features in several studies. Joe and Pyo [20] used time-domain images and frequency domain images of EEG and EOG signals to train a CNN. A fairly simple CNN architecture with only two convolutional layers attained 94 accuracy when trained with both types of image. A relatively deep CNN architecture was investigated by Yildirim et al. [21]. The model consists of 10 1-D convolutional layers that classify sleep stages from direct EEG and EOG signals. Similar to [19], in their paper, the sleep classes are also framed from two-class to six-class, finding that for single-channel EEG, EOG, or EEG-EOG combination, two-class (sleep and wake) classification accuracy was highest and remained higher than a multi-class scenario. In [22], time-frequency images obtained using Morse, Bump, and Amor continuous wavelets from a single-channel EEG signal and a pre-trained SqueezeNet model were used for sleep stage classification. When the Fpz-Cz channel was considered, scalogram images from all the wavelets resulted in accuracy slightly over 83% for 30 s epochs. When the 150 s epoch is considered, Morse wavelet scalogram provided 85.07% average accuracy. In the case of the Pz-Oz channel for 30 s epoch, Amor wavelet scalogram achieved 81.85% average accuracy, which is highest among the three scalograms. The mean accuracy increased slightly to 82.92% for the 150 s epoch when Morse wavelet scalogram is used. Zhou et al. proposed a modified CNN architecture for a single-channel EEG signal [23]. The architecture uses a multi-convolutional block to perform convolution in several stages with different filter sizes, and maximum-average pooling is used in every convolution block to maximize the feature-capturing ability. The model’s performance is presented in terms of mean accuracy for a five-fold cross-validation over two datasets. For single channel EEG C4/A1 from the Cleveland Children’s Sleep and Health Study (CCSHS) dataset, 90.2% overall accuracy is obtained. The model provided a lower average accuracy of 86.1% when the Fpz-Cz channel EEG signal is considered from the Sleep-EDF Database Expanded (Sleep-EDF) dataset. A temporal convolutional neural network (TCNN) was proposed in [24]. In that model, the TCNN segment extracts temporal features from output features generated by two parallel CNN blocks. The CNNs extract high-level features from the EEG signal, and the TCNN finds non-linear dependency patterns. The final classification is performed by a conditional random field module that provides a conditional probability for the possible sleep stages. An orthogonal CNN (OCNN) architecture was modified and its performance investigated in [25], where the authors included an alternate version of a rectified linear unit (ReLU) and the Adam optimizer in the OCNN model. The authors claimed that this modification enhances feature extraction and accuracy, and it also reduces the learning time. At the pre-processing stage, a time–frequency image is generated using empirical mode decomposition (EMD) and the Hilbert transform to train the model. This improvised model exhibited consistent performance on EEG data taken from different datasets.

A few mixed neural network architectures have been proposed for sleep stage classification. A mixed or hybrid network combines two different networks. In [26], a cascaded model comprising a CNN followed by Bidirectional Long Short Term Memory (Bi-LSTM) was proposed. Spectrograms obtained by Fourier transform are used to train the model. A multi-layer CNN is utilized to extract time and frequency features from an EEG spectrogram. Afterwards, two-layer Bi-LSTMs are employed to learn how features extracted from adjacent epochs are related to each other, and to then classify the different sleep stages. A very similar model was studied in [27], which achieved an average accuracy of 92.21% on single-channel EEG data. Another mixed architecture formed in conjugation with multilayer perceptron and an LSTM was proposed in [28] to take advantage of both sparse patterns and sequential patterns in the temporal domain of an EEG signal. This mixed model attained better accuracy than other baseline models, such as the SVM, RF, and MLP. Pei et al. proposed a combined model in [29] that conjugates a CNN and gated recurrent units (GRU) to form a sleep stage classifier. In their study, the authors considered EEG, ECG, EMG, and EOG signals for training the model simultaneously. Different stacked combinations of LSTM and Bi-LSTM structures were utilized in [30] for the same classification task. Here, a total of 24 features are computed from six EEG channels, one EOG channel, and one EMG channel in the feature extraction stage, and the best accuracy (78.90%) was reported for the LSTM/Bi-LSTM combination. A hybrid framework consisting of three different deep learning architectures was discussed in [31]. This model has two subdivisions: one is a CNN-LSTM block, and the other is a transformer network. A hidden Markov model (HMM) and a 1-D CNN were combined in [32] to form a hybrid model. The fundamental concept behind the 1-D-CNN-HMM model is to leverage the benefits of using a one-dimensional convolutional neural network to automatically extract features from raw EEG data, and then combine it with a hidden Markov model that can take advantage of prior information about sleep stage transitions between adjacent EEG epochs. The fundamental concept behind the 1D-CNN-HMM model is to leverage the benefits of using a one-dimensional convolutional neural network (1D-CNN) to extract features from raw EEG data automatically, and then combining it with a hidden Markov model (HMM) that can take advantage of the prior information about sleep stage transitions between adjacent EEG epochs. A hybrid ANN–CNN model which has similar architecture to what we investigated in this paper is studied in [33] for identifying high-risk COVID-19 patients. The ANN network is trained using clinical, demographic, and laboratory data on the other hand the CNN network is trained using computed tomography (CT) images. The authors reported an accuracy of 93.9% for the combined network. Although the paper used similar model architecture as we worked with in this paper, they used two different sources (clinical records and CT images) but in our work we utilized the same source (only the EEG signal) to create both datasets for ANN and CNN.

In most of the mixed or hybrid models studied, we observed that these models are trained with only one type of feature, like a spectrum image or raw EEG signals. Few works involved different features for training two different architectures. To explore this approach to training a hybrid model with different types of features, we implement a mixed-input model for sleep–wake state classification. The mixed-input model combines an artificial neural network (ANN) and a CNN. To train this model, two types of features are used. The ANN is trained using statistical features calculated from EEG signals that are arranged in a tabular structure, while the CNN is trained with Hilbert spectrum images. The main contributions of our research can be summarized as follows:

- A hybrid deep learning model for sleep–wake stage classification utilizing mixed features is proposed. We also describe the classification performance of our proposed model.
- We demonstrate how mixed input is used to train two different segments, and we combine their responses to finally determine the target classes.

The rest of this paper is organized as follows. In Section 3 the proposed hybrid model is briefly described. This section also explains the data preparation, and provides details of the feature extraction procedure. In Section 4, the results and discussion are presented, and concluding remarks are made in Section 5.

## 3. Methods

In this section, the architecture of our proposed model is presented. The description of the dataset and the preprocessing of the EEG signals for feature extraction is provided. Finally, the details of empirical mode decomposition (EMD) and preparing a mixed dataset from EMD coefficients are discussed.

### 3.1. Proposed Model

**Figure 1:**
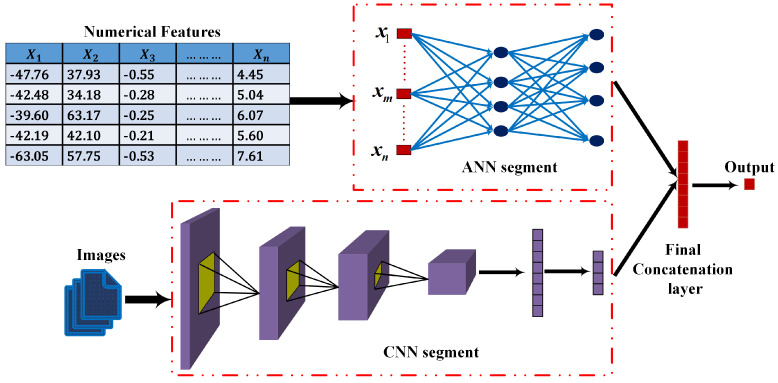
Mixed-input sleep–wake classification model.

Our proposed mixed-input ANN–CNN model is depicted in Figure 1. The CNN segment, which processes the Hilbert spectrum images, is composed of three convolutional layers. In each layer, the number of filters increases by a power of 2. Therefore, the first layer has 16 convolutional filters, and the second and third layers have 32 and 64 filters each. The kernel size is set to 3, and a rectified linear unit (ReLU) is used as the activation function in each layer. Each convolutional layer is followed by a 2×2 max pooling layer. There are two fully connected layers, one with 16 nodes and one with eight nodes, following the last convolutional layer. There is no softmax layer in the CNN network to provide a probability for the classes, unlike conventional CNNs. The output of the last fully connected layer is concatenated with the output from the ANN model.

The ANN, on the other hand, has 2 dense layers, one with 16 nodes and one with 8 nodes. The same ReLU activation is used in these nodes, and there is no softmax layer here either. Finally, the output from the CNN and the ANN are concatenated to a vector of size 16, which then proceeds to the final output through another fully connected layer of 4 nodes. Since this is a binary classification scenario, the final layer of this combined model is implemented as a sigmoid layer.

### 3.2. Data Preparation

In this work, EEG recordings from the Sleep-EDF expanded database (European Data Format) [34] are used for sleep and wake state classification. This dataset contains 197 whole-night EEG, EOG, and chin EMG recordings, and event markers. The data is collected from a total number of 77 individuals among them 40 were female and 37 were male subjects [35]. EEG recordings are collected for two nights from each individual. The mean age of the group was 54 years. The data was collected under the supervision of researchers from the Department of Neurology and Clinical Neurophysiology of Leiden University Hospital. The EEG data were collected through electrodes placed at subjects’ head at home while they were maintaining their habitual activities. A modified Oxford four-channel cassette recorder with frequency response range from 0.5 to 100 Hz is used to record the EEG. The records were converted to a digital form (12 bits/sample) in a personal computer. To check the noise levels, zero-voltage calibration signals are used which were recorded at the same time as the EEG recording.

**Figure 2:**
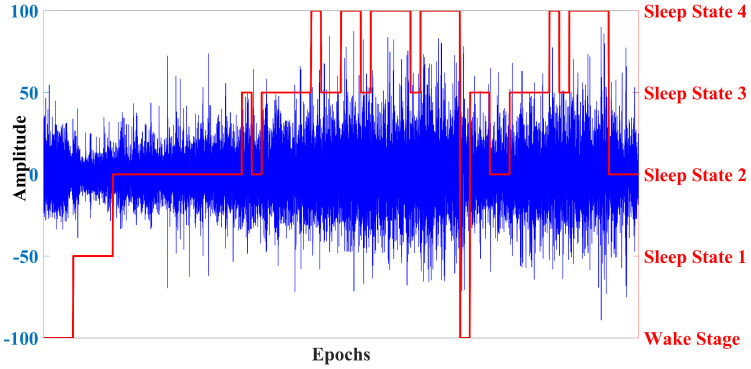
EEG signal epochs and corresponding sleep stage marker. The blue line curve represents the EEG signal for several epochs. The red line curve represents the corresponding hypnogram that represents different sleep stages.

The complete dataset contains polysomnography recordings from two studies: ***Sleep Cassette Study and Data*** and ***Sleep Telemetry Study and Data***. Although the dataset contains multiple polysomnography data, this paper only uses EEG Pz-Oz signals from ***Sleep Cassette Study and Data***. The Pz-Oz channel contains the delta wave and posterior alpha wave information. The delta wave captures the slow wave activity which is dominant in deep sleep stages whereas the alpha waves are generally observed in relaxed wakefulness state and light sleep states. Our goal was to develop a sleep/wake state classification with a single channel EEG data. Thus, we choose the Pz-Oz signal. The Sleep-Cassette data include 153 sleep recordings from healthy Caucasian individuals with ages ranging between 25 and 101. EEG signals were sampled at 100 Hz. The dataset also contains annotation information that corresponds to the physiological signals. These annotation files are called ***Hypnograms***. The hypnogram contains sleep patterns for each 30-s recording. This 30-s duration is an epoch, which is a standardized format for data analysis in sleep-related research. This sort of segmented recording allows for identification and classification of different sleep stages based on the pattern of the physiological signals observed during each epoch. This is also beneficial for efficient data storage and retrieval. The sleep patterns marked in the hypnogram file consist of the following sleep stages: W (wake state), REM, 1, 2, 3, 4, M (movement time), and ? (not scored). All hypnograms were manually evaluated by skilled medical technicians according to the Rechtschaffen and Kales manual. In our analysis we discarded recordings corresponding to movement time (M) and not scored (?). A visual representation of the EEG signal and its concurrent hypnogram patterns is presented in Figure 2.

In Figure 2, EEG signals for several epochs are shown as the blue line plot, and the respective stage of sleep in that epoch is presented by the multi-labeled red line. Clearly, the EEG signal corresponds to several stages, but our focus is to build a model that can classify two sleep stages: the wake state and the sleep state. To frame this multi-sleep-state scenario into a binary-state scenario, we proceed by considering the five sleep stages (from sleep stage 1 to sleep stage 4, plus REM) as a single class (the sleep class) and the remainder represents the wake class. However, this approach leads to an imbalanced dataset where more samples correspond to the sleep class than to the wake class. To address this issue, an equal number of random samples were removed from the sleep instances to balance the two classes in a restructured dataset. Therefore, the data preparation has two steps:

1. Merge the different sleep stages into a single sleep class.
2. Perform random sampling to balance the classes in the dataset.

The abovementioned process created the dataset used in this work. The size of the original dataset is quite large, so for this work, EEG recordings of from four individuals were used to construct a fairly sized dataset to proceed with. The final dataset consisted of 6880 epochs with equal instances of both classes.

### 3.3. Feature Extraction

As described previously, our proposed model process both tabular and image features to perform classification. In this section, we discuss extraction of both types of feature.

Using time frequency analysis tools to compute features from bio-signals (especially EEG signals) is widely adopted by researchers, and it has shown great performance in many applications. Following that, we utilized the EMD approach (a powerful technique for analyzing non-stationary signals) to calculate the features. EMD offers several advantages such as more detailed time-frequency analysis than STFT and WT. STFT uses a fixed window size, which limits its time-frequency resolution, whereas WT uses a fixed set of basis functions, which limits its ability to capture the local features of a signal. EMD, on the other hand, adaptively decomposes a signal into its Intrinsic Mode Functions (IMFs), which provides a more detailed and accurate time-frequency representation of the signal. Additionally, EMD does not suffer from the Heisenberg uncertainty principle because it is an adaptive technique that can adjust its time and frequency resolution based on the local features of the signal. Finally, EMD can be used to analyze a wide range of signals without the need for specific assumptions about the signal’s underlying properties. In contrast, STFT and WT require prior selection of the window size or basis functions, respectively, which may not always be optimal for the signal being analyzed.

In the EMD process, a non-stationary signal, x(t) is decomposed into a set of intrinsic mode functions, $h_{i}\left(t\right)$, and a residual $r_{n}\left(t\right)$:

(1) $$x(t)=\sum_{i=1}^{N}h_{i}\left(t\right)+r_{n}\left(t\right),$$

The IMFs are computed using an iterative process as follows

(i) Compute the upper and lower envelopes, u(t) and l(t), by interpolating the local maxima and minima of the signal using cubic-spline interpolation.
(ii) Compute the mean of upper and lower envelopes: $m(t)=\frac{u(t)+l(t)}{2}$
(iii) Compute the difference between the signal and the mean envelope: d(t)=x(t)−m(t)
(iv) If d(t) is a monotonic function, then $h_{i}\left(t\right)=d(t)$ and the algorithm terminates. Otherwise, repeat steps (i)–(iii) on d(t) to obtain the next IMF.

An IMF satisfies two conditions:

(a) The number of extrema (maxima and minima) and the number of zero-crossings must be equal or differ by at most 1.
(b) The mean value of the function over any complete cycle must be zero.

The pseudocode for computing the IMFs are presented in Table 1.

The IMFs can be used to analyze the frequency content of the signal as a function of time. The first IMF typically represents the highest frequency oscillatory mode in the signal, whereas the last IMF represents the lowest frequency mode. The number of IMFs obtained from a signal depends on its complexity and the desired level of decomposition. Because IMFs represent various frequency contents from the complex signal, in this work, we compute statistical features from the IMFs obtained from the epochs of the EEG recordings to create the numerical features to be used in the mixed-input model in tabular format. Statistical features are listed in Table 2 with the equations to compute them.

**Figure 3:**
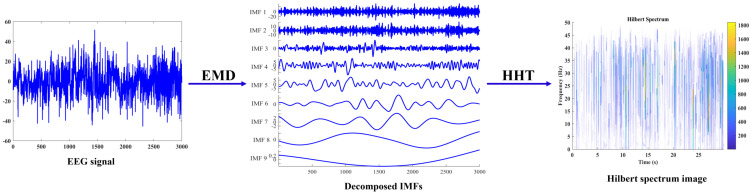
Hilbert spectrum image generation process. The EEG epochs (a sample epoch is presented in the left plot) are decomposed using EMD which results in corresponding IMFs (shown in the middle plot). Finally, using HHT a Hilbert spectrum is obtained from the IMFs (shown in the right plot).

There is a CNN segment in the proposed mixed-input model, so the other feature to be used in the mixed-input model is images. Many researchers utilize spectrogram images to analyze various bio-signals by observing the spectral content of the signal by time or frequency. Common spectrograms are obtained through various time-frequency analysis methods like STFT and WT. In computing the first type of feature, we used EMD and the resulting IMFs to calculate numerical features. We think it logical to use the same tool to obtain a spectrogram using the same decomposition technique, and to use the spectrogram image as the image feature for our model. Therefore, Hilbert spectrum images are obtained from IMFs resulting from the EMD by using the Hilbert–Huang transform (HHT), and the spectrum image is used as the feature to be processed by the CNN component of the model. The Hilbert spectrum, unlike a traditional spectrogram, can capture and analyze frequency modulations and transients that may be missed by other spectral analysis methods [36,37]. The process of obtaining the Hilbert spectrum is visually depicted in Figure 3.

**Figure 4:**
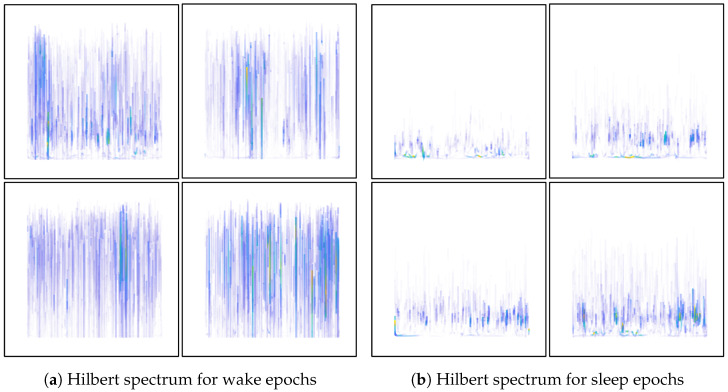
Samples of Hilbert spectrum images created from sleep and wake EEG epochs following the process showed in Figure 3.

At the end of the feature extraction stage, two types of feature are now available: one is a numerical dataset in tabular format, and the other is an image dataset that contains Hilbert spectrum images for two classes, namely, the wake and sleep stages. In both datasets, there is an equal number of instances for the two classes. Some sample Hilbert spectrum images for the two classes are provided in Figure 4a,b.

## 4. Results and Discussion

In this section, the classification performance of the proposed mixed-input model is discussed. To assess the classification performance of a model, a confusion matrix is used. It summarizes the number of correct and incorrect predictions made by the model by comparing predicted classes with actual classes. In a binary classification framework, the confusion matrix has four entries: true positive (*TP*), false positive (*FP*), true negative (*TN*), and false negative (*FN*). In the present classification problem, they can be interpreted as follows:

- ***TP*****:** the number of samples correctly classified as the wake class.
- ***FP*****:** the number of sleep class samples incorrectly predicted as the wake class.
- ***TN*****:** the number of samples correctly classified as the sleep class.
- ***FN*****:** the number of wake class samples incorrectly predicted as the sleep class.

These are used to compute the following performance metrics:

(2) $$Accuracy=\frac{TN+TP}{TN+FP+TP+FN}$$

(3) $$Precision=\frac{TP}{FP+TP}$$

(4) $$Recall=\frac{TP}{FN+TP}$$

(5) $$F1-score=\frac{TP}{TP+\frac{1}{2}\left(FP+FN\right)}$$

*Accuracy* is a general measure of the model’s overall correctness calculated by dividing the number of correct predictions (true positives and true negatives) by the total number of instances in the dataset. *Precision* indicates reliability of the model when it predicts a positive outcome. A higher precision score indicates fewer false positives, which means the model is more accurate in identifying positive instances. On the other hand, *Recall* evaluates the model’s ability to find all positive instances. A higher recall score indicates a lower rate of false negatives, meaning the model is better at capturing positive instances. Finally, The *F1-score* is the harmonic mean of precision and recall. It provides a balance between precision and recall, considering both the quality and completeness of the predictions. The best possible value for *F1-score* is 1. The *F1-score* penalizes models that have a significant difference between precision and recall.

**Figure 5:**
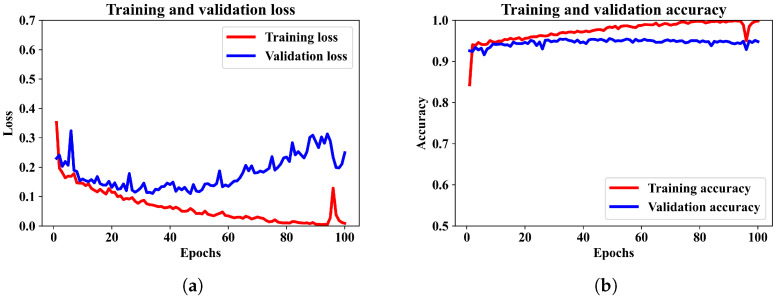
Training and validation performance of CNN model only. (**a**) shows the training and validation loss. (**b**) shows the training and validation accuracy over the training epochs for the CNN model.

Initially, the classification performance is observed utilizing only a CNN model. The CNN model has the same architecture as the CNN segment described in the proposed model in Section 3.1. The CNN model is compiled and trained using similar optimizer, loss function and batch sizes as it for the mixed-input ANN–CNN model. The training and validation performance of the CNN model after same number of epochs are presented in Figure 5a,b.

**Figure 6:**
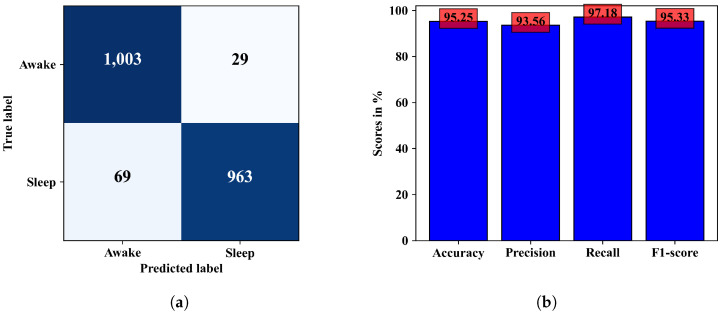
Performance metrics of the CNN model. (**a**) shows the confusion matrix and (**b**) shows the accuracy, precision, recall, and F1-score for the CNN model.

From the training and validation loss pattern in Figure 5a it can be observed that the validation loss has an increasing pattern but the training loss continues to decrease. It means there is a minor case of overfitting the model. The CNN model attained an average accuracy of 95.25%. The corresponding confusion matrix and other performance metrics are presented in Figure 6a,b.

**Figure 7:**
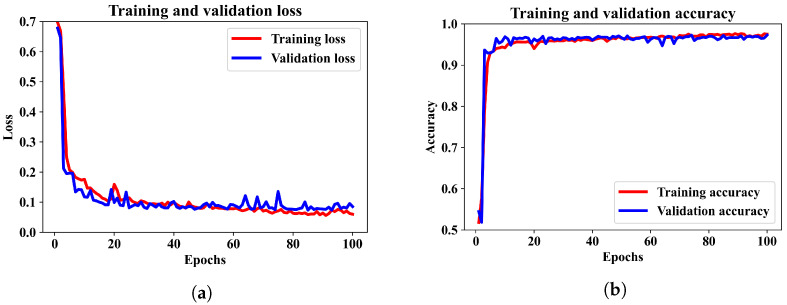
Training and validation performance of the proposed model. (**a**) shows the training and validation loss. (**b**) shows the training and validation accuracy over the training epochs.

Finally, we included the numerical features and Hilbert spectrum images to train the combined ANN–CNN model. The training and validation performance of the proposed model are illustrated by the loss and accuracy curves in Figure 7a,b.

We can see that the model was trained for 100 epochs, and the patterns for binary cross-entropy loss and accuracy in the training and validation data have regular patterns. The loss was reduced sharply until epoch 10 and stabilized after epoch 20. Similarly, the accuracy converged to around 96% after 60 epochs. It has been observed that when the model is trained for more than 100 epochs, the accuracy did not increase. Therefore, for this model an epoch 100 can be considered as optimal.

**Figure 8:**
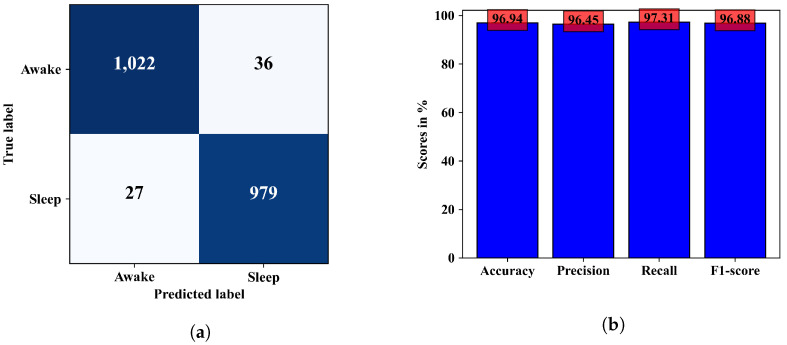
Performance metrics of the mixed-input model. (**a**) shows the confusion matrix and (**b**) shows the accuracy, precision, recall, and F1-score for the mixed-input model.

The ability of the model in classifying EEG epochs for a test dataset can be understood better from the confusion matrix in Figure 8a, where 2001 of 2064 test samples were correctly classified, and the model was slightly better at predicting the wake class than the sleep class. The other performance metrics calculated from this confusion matrix are in Figure 8b. Observing the confusion matrices in Figure 6a and Figure 8a, we can see that the combined model could classify a few more samples better than the CNN model only. In terms of accuracy the combined ANN–CNN model has 1.69% higher accuracy than the CNN model. Although the improvement is not significantly large, the ANN–CNN model can be considered more stable than the CNN model as there is small overfitting issue present in the CNN model as seem from the training and validation loss in Figure 5a.

**Figure 9:**
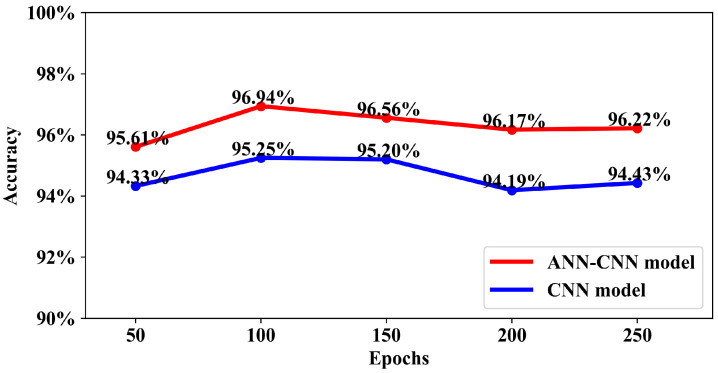
Effect of training the ANN–CNN and CNN model for different number of epochs.

In Figure 9, the effect of training both the models for different number of epochs is presented. From the line plots, it can be observed that both the models have their best classification accuracy when they are trained for 100 epochs which are 96.94% and 95.25% for ANN–CNN and CNN model respectively. For the combined ANN–CNN model the accuracy stays around 96% even it is trained for more than 100 epochs. In case of the CNN model, the model accuracy decreases to around 94% for more training epochs. Observing the pattern in the plot, the training the model for 100 epochs would be appropriate.

The performance of the proposed hybrid model is also observed for classifying the all available sleep stages in the dataset. There are six sleep stages corresponding to the EEG epochs: wake, sleep stage 1, sleep stage 2, sleep stage 3, sleep stage 4 and REM sleep stage. EEG epochs marked with Movement time (M) and not scored (?) are discarded in the data preparation stage. In total, 5440 samples/EEG epochs are considered from four subjects according to the distributions listed in Table 3.

**Figure 10:**
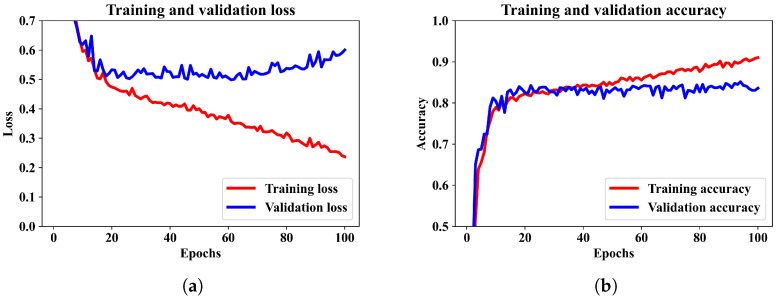
Training and validation performance of the proposed model for six-class classification scenario. (**a**) shows the training and validation loss. (**b**) shows the training and validation accuracy.

Although, there are more samples in the sleep class since more epochs in the dataset belongs to wake state, 2000 wake samples are considered so that the wake instances do not outnumber the other class samples. Even after down-sampling the wake class, the sleep stage 1, 3, and 4 still have relatively lower number of samples compared to the remaining three classes. With this sample distribution, the ANN–CNN model is trained for 100 epochs as numbers from Figure 9 shows that the model could achieve best accuracy for 100 epochs. The loss and accuracy trends over the training period are presented in Figure 10a,b.

**Figure 11:**
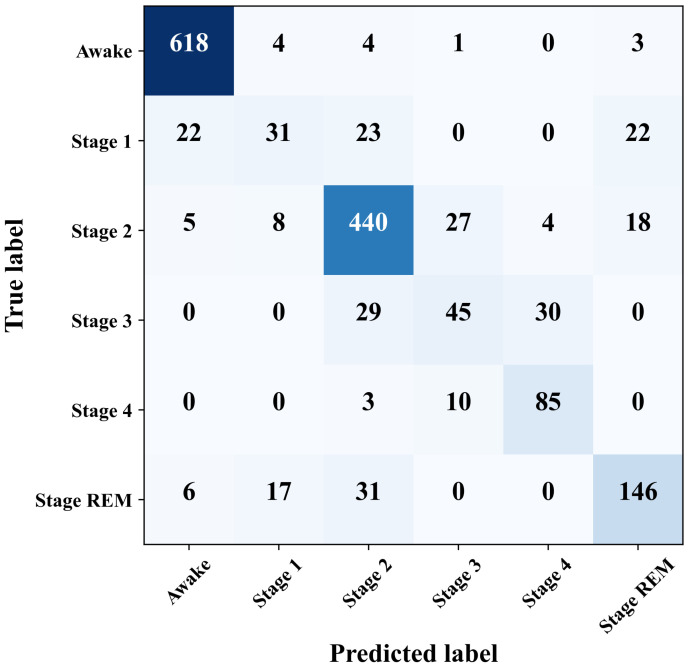
Confusion matrix of the ANN–CNN model for six-class sleep stage classification.

The trend in training and validation losses in Figure 10a is a manifestation of over-fitting. The maximum validation accuracy attained by the model is 83.64% for six-class sleep state classification. The performance of the model in classifying each individual classes can be better realized from the confusion matrix depicted in Figure 11.

The numbers in the confusion matrix indicates that the model is quite good at classifying the wake class and shows somewhat descent performance in detecting sleep stages 2, 4, and REM sleep stage. However, the model is not good at distinguishing sleep stages 1 and 3. If we consider the numbers in Table 3, it shows that these two sleep stages along with sleep stage 4 have lower number of samples. This is one reason that the model could not classify these sleep stages with good accuracy. Another possible reason could be the image pattern of the Hilbert spectrum. The Hilbert spectrum image for the wake class has quite distinctive pattern than the images of the remaining classes. Besides having lower number of samples, the Hilbert spectrum images of sleep stage 1, 3, 4, and REM stage may have a highly similar pattern which results less accurate classification for those classes. A class-wise list of precision, recall, and F1-score is provided in Table 4.

Although the ANN–CNN model does not perform equally well for all the classes, it still has promising performance even with similar patterns in the Hilbert transform image. This model might demonstrate better classification if different image encoding from the EEG signals is utilized which generates more distinctive images for different sleep stage EEG recordings.

The performance of the mixed-input model in terms of classification accuracy is compared in Table 5 with some existing work. Most of the work considered here used Sleep EDF or Sleep EDF Extended (EDFx) datasets and a combined deep learning architecture. Three works were based on different datasets; University College Dublin Sleep Apnoea Database (UCDDB), Sleep Heart Health Study (SHHS), and Massachusetts Institute of Technology and Beth Israel Hospital (MIT-BIH) are included because they used hybrid classification models. In most of the work, multiple sleep classes are classified, and in some work, two-class accuracy (sleep and wake) is reported. Two-class accuracy scores are listed in Table 5 when the research address 2–5 class classification.

As we can observe in the table, most of the hybrid model is implemented for classifying five sleep stages and the works thus addressed do not use hybrid models. Although the results of two-class classification in other work seem better than our proposed method, they mostly used advanced feature extraction approaches. As in [40,43], the classification accuracy is better than our model, but they used advanced approaches as Complete Ensemble Empirical Mode Decomposition with Adaptive Noise (CEEMDAN) and Ensemble Empirical Mode Decomposition (EEMD) along with spectral features. Ronzhina, M. et al. [42] also attained better accuracy in case of two-class classification. In this work the authors utilized more than one channel signal and numerous features from time, frequency, time–frequency, nonlinear, and hybrid features. The 1-D CNN model proposed in [21] achieved higher accuracy by using combinations of EEG, EOG, and EEG + EOG combined signals. Additionally, the proposed 1-D CNN model is very deep and consists of 10 convolutional layers. Therefore, if more complex means of feature extraction or very deep models are utilized, the classification accuracy can be improved. In our work, we tried to keep the feature extraction process less complex and we created a mixed feature for both CNN and ANN segments of the hybrid model. We used the same EMD coefficients for calculating the numerical features, less complex to calculate, for ANN and the image features for CNN segments. We think, due to this trade-off, our accuracy is somewhat less than other two-class classification tasks. In future work, we will focus on extracting more effective numerical features and implement different image encoding approaches for creating image datasets from the EEG signal to ensure better distinction among different class samples. Also, augmenting images to balance the dataset will be considered.

## 5. Conclusions

In this paper, a hybrid model is proposed for sleep and wake state classification from single-channel EEG signals. The proposed model combines ANN and CNN models by concatenating the output from the fully connected layer of the two architectures. The proposed model is trained using mixed-input with statistical features computed after performing EMD on EEG signals and by obtaining Hilbert spectrum images. The statistical features, organized in tabular format, are processed by the ANN, and the images are processed by the CNN. Single-channel EEG signals collected from the Sleep-EDF expanded database and the EEG epoch labels are reorganized to create a dataset having two classes: sleep and wake. As a primary approach, the model was trained with EEG signals from four random individuals, and after training the model for 100 epochs, the highest accuracy, **96.94%**, was achieved, which is promising because of the simple statistical features considered. In future, we plan to address the classification of multiple sleep stages with more advanced features, and will include all subjects’ EEG signals from the Sleep-EDF expanded database.

## Figures and Tables

**Table 1:** Pseudocode for computing IMFs.

**Input:** Signal x(t)
**Output:** IMFs
**Initialize residue: $r_{n}\left(t\right)$** = x(t)
**Initialize IMFs** = [ ]
**while** $r_{n}\left(t\right)$ has more than one extrema **do:**
Find all local extrema in $r_{n}\left(t\right)$.
Initialize upper envelope, u(t) by connecting local maxima with cubic spline.
Initialize upper envelope, l(t) by connecting local maxima with cubic spline.
Compute the mean of upper and lower envelopes, $m(t)=\frac{u(t)+l(t)}{2}$.
Append the current IMF to **IMFs**.
Update residue, $r_{n}\left(t\right)$ by subtracting the current IMF from it.
Append the final residue to **IMFs**.
return **IMFs**.

**Table 2:** Statistical features and their equations.

Feature	Equation	Feature	Equation
Minimum	$\min\left(y_{i}\right)$	Maximum	$\max\left(y_{i}\right)$
Mean	$\mu =\frac{1}{N}\sum_{i=1}^{N}y_{i}$	Standard deviation	$\sigma =\sqrt{\frac{\sum_{i=1}^{N}{\left(y_{i}-\mu \right)}^{2}}{N}}$
Kurtosis	$K=\frac{1}{N}\sum_{i=1}^{N}\frac{{\left(y_{i}-\mu \right)}^{4}}{\sigma^{4}}$	Skewness	$S=\frac{1}{N}\sum_{i=1}^{N}\frac{{\left(y_{i}-\mu \right)}^{3}}{\sigma^{3}}$
Root Mean Square	$y_{RMS}=\sqrt{\frac{1}{N}\sum_{i=1}^{N}\left|{y_{i}}^{2}\right|}$	Crest factor	$CF=\frac{max(y_{i})}{{y_{i}}_{RMS}}$
Shape factor	$SF=\frac{y_{RMS}}{\frac{1}{N}\sum_{i=1}^{N}\left|y_{i}\right|}$	Impulse factor	$IF=\frac{y_{peak}}{\frac{1}{N}\sum_{i=1}^{N}\left|y_{i}\right|}$
Clearance factor	$CLF=\frac{y_{peak}}{{\left(\frac{1}{N}\sum_{i=1}^{N}\sqrt{\left|y_{i}\right|}\right)}^{2}}$	Variance	$S^{2}=\frac{\sum_{i=1}^{N}\left(y_{i}-\mu \right)}{n-1}$
Energy	$E=\sum_{n=-\infty }^{\infty }{\left|y_{n}\right|}^{2}$	Power	$P=\lim_{N\to \infty }\frac{1}{N}\sum_{n=0}^{n=N-1}{\left|y\left(n\right)\right|}^{2}$
Peak to RMS	$\frac{y_{peak}}{y_{rms}}$	Range	$y_{max}-y_{min}$

**Table 3:** Number of samples in different classes.

Class	Number of Samples
Wake stage	2000
Sleep stage 1	320
Sleep stage 2	1730
Sleep stage 3	364
Sleep stage 4	353
Sleep stage REM	673

**Table 4:** Class-wise precision, recall, and F1-scores.

Class	Precision	Recall	F1-Score
Wake stage	94.93%	98.09%	96.49%
Sleep stage 1	51.67%	31.63%	39.24%
Sleep stage 2	83.02%	87.49%	85.27%
Sleep stage 3	54.21%	43.27%	48.13%
Sleep stage 4	71.43%	86.73%	78.34%
Sleep stage REM	77.25%	73.00%	75.06%

**Table 5:** Comparison with similar works on relevant datasets.

Study	Dataset	Methods	Number of Classes	Accuracy
Li et al. [26]	Sleep EDF and EDFx	CNN-Bi-LSTM	5	94.17%
Joe, M.J. and Pyo, S.C. [20]	Sleep EDFx	CNN	4	94%
jadhav, P. et al. [22]	SleepEDFx	SqueezeNet	5	<84%
Yildirim, O. et al. [21]	Sleep EDF and EDFx	1-D CNN	2–6	98.33%
Jiang, D. et al. [38]	Sleep EDF and EDFx	Hidden Markov Model	5	92.7%
Pei, W. et al. [29]	UCDDB and SHHS	CNN-GRU	5	68.28% to 83.15%
Hao, J. et al. [27]	MIT-BIH	CNN-Bi-LSTM	5	92.21%
Kuo, C.E. and Chen, G.T. [30]	PhysioNet2018	Hybrid stacked LSTM	5	83.07%
Yang, B. et al. [32]	Sleep EDFx	1-D-CNN-HMM	5	83.23%
Hassan, A.R. et al. [39]	Sleep EDF	Bagging(Decision tree)	2–5	95.05%
Hassan, A.R. and Bhuiyan, M.I.H. [40]	Sleep EDF	CEEMDAN-Bagging	2–6	99.48%
Berthomier, C. et al. [41]	Proprietary dataset	Fuzzy classification	2–5	96%
Ronzhina, M. et al. [42]	Sleep EDF	ANN	2–6	96.9%
Hassan, A.R. and Bhuiyan, M.I.H. [43]	Sleep EDF	EEMD-Random under sampling boosting	2–6	98.15%
**Proposed method**	**Sleep EDFx**	**ANN–CNN**	**2, 6**	**96.94%, 83.64%**

## Data Availability

Not applicable.
